# Relationship Between L-DOPA-Induced Reduction in Motor and Exploratory Activity and Striatal Dopamine D_2_ Receptor Binding in the Rat

**DOI:** 10.3389/fnbeh.2015.00352

**Published:** 2016-01-06

**Authors:** Susanne Nikolaus, Markus Beu, Maria A. de Souza Silva, Joseph P. Huston, Hubertus Hautzel, Claudia Mattern, Christina Antke, Hans-Wilhelm Müller

**Affiliations:** ^1^Clinic of Nuclear Medicine, University Hospital DüsseldorfDüsseldorf, Germany; ^2^Center for Behavioural Neuroscience, Institute of Experimental Psychology, Heinrich-Heine UniversityDüsseldorf, Germany; ^3^M et P Pharma AGEmmetten, Switzerland; ^4^Oceanographic Center, Nova Southeastern UniversityFort Lauderdale, FL, USA

**Keywords:** D_2_ receptor, [^123^I]IBZM, L-DOPA methylester, motor behavior, exploratory behavior, habituation, time-behavior curves, small animal SPECT

## Abstract

**Purpose:** The present study assessed the influence of L-DOPA administration on neostriatal dopamine (DA) D_2_ receptor binding in relation to motor and exploratory behaviors in the rat.

**Methods:** D_2_ receptor binding was measured in baseline, after challenge with the aromatic L-amino acid decarboxylase inhibitor benserazide, and after challenge with either 5 or 10 mg/kg L-DOPA plus benserazide. Additional rats received injections of saline. For baseline and challenges, striatal equilibrium ratios (V${}_{3}^{\prime \prime }$) were computed as estimation of the binding potential. Motor and exploratory behaviors were assessed for 30 min in an open field prior to administration of [^123^I]IBZM. D_2_ receptor binding was measured with small animal SPECT 2 h after radioligand administration for 60 min.

**Results:** Both L-DOPA doses significantly reduced D_2_ receptor binding relative to baseline and led to significantly less ambulation, less head-shoulder motility, and more sitting relative to saline. Moreover, 10 mg/kg L-DOPA induced less head-shoulder motility, more sitting, and more grooming than 5 mg/kg L-DOPA. Analysis of time-behavior curves showed that L-DOPA-treated animals relative to saline exhibited a *faster* rate of decrease of ambulation frequency and a *slower* rate of decrease of both duration and frequency of head-shoulder motility from a *lower* maximum level.

**Conclusions:** The reductions of striatal D_2_ receptor binding after L-DOPA may be conceived to reflect elevated concentrations of synaptic DA. L-DOPA-treated animals showed less ambulation and less head-shoulder motility than saline-treated animals, indicating an association between less behavioral activity and increased availability of striatal DA. The *faster* rate of decrease of ambulation frequency and the *lower* maximum levels of both head-shoulder motility duration and frequency may be interpreted in terms of influence of increased DA availability on behavioral habituation to a novel environment.

## Introduction

Deficiencies of D_2_ receptor function are associated with a variety of psychiatric conditions including anxiety disorder, major depressive disorder, and schizophrenia (for reviews see Nikolaus et al., 2010, 2012, 2014a) as well as numerous neurological diseases such as M. Huntington, multiple system atrophy, progressive supranuclear palsy, and late-stage idiopathic Parkinson's disease (PD, for review see Nikolaus et al., 2009).

Idiopathic PD may be effectively treated with the dopamine (DA) precursor L-3,4-dihydroxyphenylalanine (L-DOPA; for review see Okereke, 2002). Initially, the increased availability of DA compensates for the decline of DA synthesis, DA storage, and DA transporter (DAT) binding, which are characteristic for idiopathic PD. With extended L-DOPA intake, however, inhibitory feedback mechanisms exerted by presynaptic D_2_ receptor binding sites lead to a reduction of endogenous DA formation, resulting in an aggravation of DAergic depletion. Common features of long-term treatment with L-DOPA, therefore, are dyskinesias and on-off and wearing-off phenomena (for review see Cenci et al., 2011).

So far, there are four small animal imaging studies, which assessed striatal D_2_ receptor binding after L-DOPA in healthy (Opacka-Juffry et al., 1998) or 6-hydoxydopamine(6-OHDA)-lesioned rats (Hume et al., 1995; Sossi et al., 2009; Sahin et al., 2014). In healthy animals, challenge with 20 or 100 mg/kg L-DOPA plus 25 mg/kg carbidopa led to a significant 40% increase of striatal [^11^C]raclopride binding relative to untreated controls (Opacka-Juffry et al., 1998). This effect was also observed when the central amino acid decarboxylase (AADC) inhibitor m-hydroxybenzylhydrazine (NSD-1015; 100 mg/kg) was given in addition to the peripheral AADC inhibitor carbidopa. It was abolished, however, when chronic L-DOPA pre-treatment (170 mg/kg/day for 5 weeks) preceded the acute challenge.

In 6-OHDA-lesioned rats, challenge with 20 mg/kg L-DOPA plus 25 mg/kg carbidopa and 100 mg/kg NSD-1015 significantly increased [^11^C]raclopride binding in the lesioned striatum relative to the contralateral side and to unlesioned controls by 16 and 23%, respectively. However, also in the unlesioned striatum, 20 mg/kg L-DOPA plus 25 mg/kg carbidopa as well as 100 mg/kg NSD-1015 led to elevations of [^11^C]raclopride binding by 17 and 25%, respectively, relative to unlesioned controls (Hume et al., 1995). This is contrasted by the findings of Sossi et al. (2009), who observed a ≈5% reduction of [^11^C]raclopride binding relative to baseline (significance not determined) in the lesioned striatum after treatment with 50 mg/kg L-DOPA plus 15 mg/kg benserazide. Similarly, Sahin et al. (2014) reported a 4% reduction of [^18^F]fallypride binding relative to baseline in the lesioned striatum after challenging 6-OHDA-lesioned rats under long-term treatment with 6 mg/kg/day L-DOPA plus 10 mg/kg/day benserazide for 4 weeks (withdrawal time: 2 days) with a single dose of 12 mg/kg L-DOPA (no reference to AADC inhibition). In neither study was L-DOPA challenge found to reduce radioligand binding relative to baseline in the contralateral striatum.

In rodents, L-DOPA is known to affect motor behaviors dependent on the administed dose and the age of animals: while lower doses (60–400 mg/kg) have been found to decrease motor activity, high doses (≥500 mg/kg) lead to an enhancement of motor activity (Boissier and Simon, 1966; Strömberg, 1970; Bryson and Bischoff, 1971; Gronan, 1975). Moreover, an increase of motor activity was observed in neonatal (5- to 8-day-old; McDevitt and Setler, 1981) and immature (18- to 20-day-old) rats (Grigoriadis et al., 1996) after doses of 12.5–50 and 150 mg/kg, respectively, whereas motor activity was decreased at 25–30 days of age (Grigoriadis et al., 1996). In previous *in vivo* imaging studies we have shown that L-DOPA doses of 5 and 10 mg/kg reduce [^123^I]FP-CIT binding to the striatal DA transporter (DAT) and that decreases and increases of DAT binding may be related to increases and decreases, respectively, of motor and exploratory activity (Nikolaus et al., 2013, 2014b).

In the present study, we set out to complement these findings by assessing the effect of 5 and 10 mg/kg L-DOPA on behavior and on D_2_ receptor binding in the rat striatum. In order to allow comparisons between treatment groups, behavior, and D_2_ receptor binding was also assessed in saline-treated rats. D_2_ receptor binding data in baseline and after challenge with 5 and 10 mg/kg L-DOPA was used to estimate DA release. Moreover, temporal dynamics of behavior were assessed by fitting suitable models to the acquired data and by statistically comparing time–behavior (*t–b*) curves (Nikolaus et al., 2014b).

## Materials and methods

### Animals

**Figure 1:**
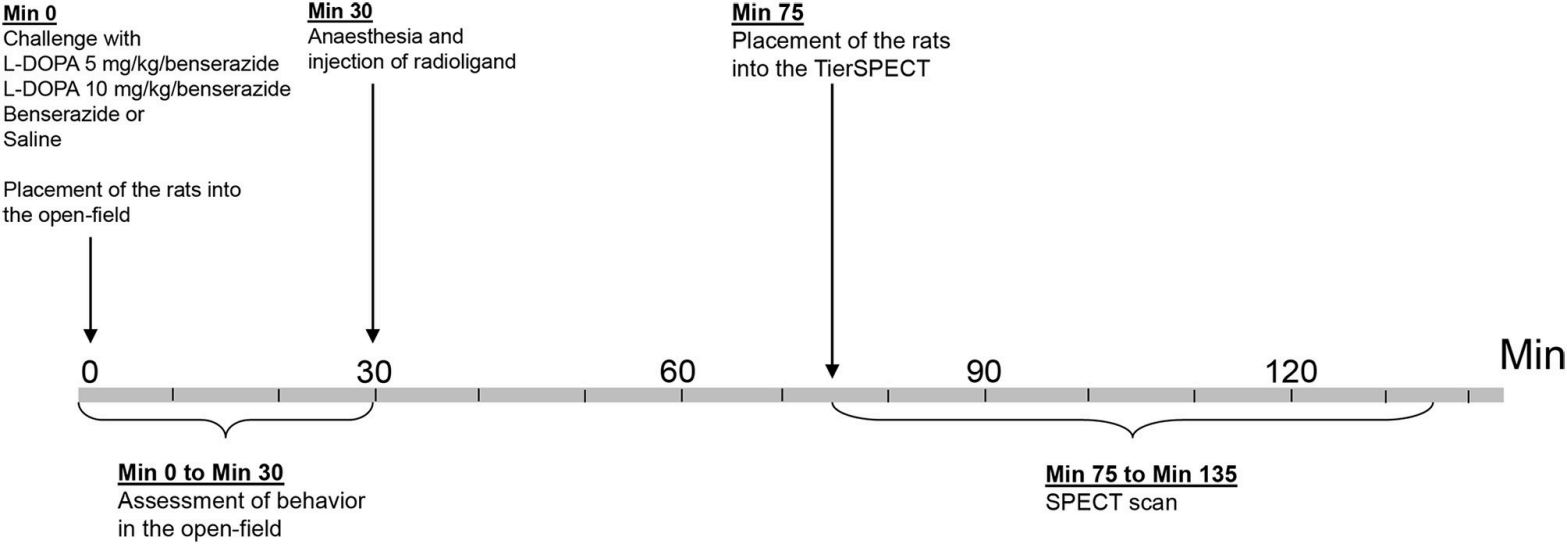
**Timeline of experimental procedure**.

The present study employed a total of 48 adult male Wistar rats (ZETT, Heinrich-Heine University, Düsseldorf, Germany), weighing 411 ± 43 g (mean ± SD). A total of 33 animals underwent D_2_ receptor imaging studies in baseline, after treatment with the aromatic AADC inhibitor benserazide and/or after treatment with either 5 or 10 mg/kg L-DOPA plus benserazide. Motor and exploratory behaviors were assessed contingent on adminsitration of either 5 or 10 mg/kg L-DOPA/benserazide (Figure 1). Five animals (5 mg/kg L-DOPA: *n* = 1; 10 mg/kg L-DOPA: *n* = 4) merely underwent behavioral measurements without imaging, since they dropped out after administation of anesthesia. Sixteen further rats underwent behavioral measurements and assessment of D_2_ receptor binding after treatment with vehicle (0.9% saline). Also here, two animals dropped out after administation of anesthesia, and, therefore, merely underwent behavioral testing. Rats were maintained in standard macrolon cages (590 × 380 × 200 mm; three animals per cage) in a climate cabinet (Scantainer, Scanbur BK, Karslunde, Denmark; temperature, 20°C; air humidity, 70%) with an artificial ligh-dark cycle (lights on at 6:00 a.m., lights off at 6:00 p.m.) and food and water freely available. The study was approved by the regional authority and carried out in accordance with the “Principles of laboratory animal care” (NIH publication No. 86-23, revised 1985) and the German Law on the Protection of Animals.

### SPECT camera

The small animal tomograph (“TierSPECT”) was described in detail elsewhere (Schramm et al., 2000). For ^123^I, tomographic resolution and sensitivity amounted to 3.4 mm and 16 cps/MBq, respectively. A low-energy ultra-high-resolution parallel-hole collimator (LEUHR, 37 × 1 × 0.2 mm^3^) was mounted in front of the detector head. Data were acquired in a 128 × 128 matrix with a pixel width and a slice thickness of ≈0.664 mm, respectively. Acquisition was conducted for 60 min in a step-and-shoot mode over a circular orbit in angular steps of 6° (60 projections, 60 s/projection). Data were reconstructed with an iterative ordered-subset-expectation-maximization algorithm (three iterations, four subsets/iteration). No post-filtering procedure was applied. An attenuation correction of 0.11 cm^−1^ was implemented assuming a uniformly attenuating medium.

### D_2_ receptor imaging studies

D_2_ receptor binding was assessed in baseline, after benserazide (Sigma-Aldrich, Taufkirchen, Germany; dose: 10 mg/kg, concentration: 10 mg/ml), after L-DOPA methylester (Sigma-Aldrich, Taufkirchen, Germany; dose: 5 and 10 mg/kg, concentrations: 5 and 10 mg/ml) plus benserazide (dose, 10 mg/kg; concentration, 10 mg/ml) and after vehicle (0.9% saline; B. Braun Melsungen AG, Melsungen, Germany; dose: 1 ml/kg). Benserazide is a peripherally-acting AADC inhibitor, which is applied in order to prevent the metabolisation of L-DOPA before it passes the blood-brain barrier (Shen et al., 2003).

Since maximum striatal DA concentrations are reached 40 min after i.p. L-DOPA and remain stable for ~2 h (e.g., de Souza Silva et al., 1997), challenges were applied intraperitoneally (i.p.) 30 min prior to radioligand application and 75 min prior to the beginning of SPECT acquisition. L-DOPA and benserazide were administed simultaneously, since pre-treatment with benserazide up to 3 h prior to L-DOPA administration did not alter motor responses to L-DOPA compared to simultaneous application of both compounds (Tayarani-Binazir et al., 2010). Thirty minutes after benserazide, L-DOPA/benserazide, or saline, animals received i.p. injections of 0.9 ml/kg ketaminehydrochloride (Ketavet®, Pharmacia GmbH, Erlangen, Germany; concentration: 100 mg/ml) and 0.4 ml/kg xylazinehydrochloride (Rompun® Bayer, Leverkusen, Germany; concentration: 2%). Then 26 ± 4 MBq [^123^I]IBZM (GE Healthcare, München, Germany; concentration: 3.4 × 10^−9^ g/ml, specific activity: >74 TBq/mmol at reference time) were injected into the lateral tail vein using a winged infusion needle set. The tube was rinsed with 1 ml 0.9% saline amounting to a total injection volume of 1.3 ml.

Since previous studies on both humans and rats had shown that specific binding of [^123^I]IBZM in the striatum reaches a plateau at about 40 min post-injection, which remains stable for up to 2 h (Verhoeff et al., 1991; Seibyl et al., 1992), SPECT measurements of D_2_ receptor binding were started 45 min after radioligand administration. Since SPECT measurements were conducted over 1 h, animals were kept under anesthesia for a total of 105 min.

### Behavioral studies

Immediately after the injection of 5 or 10 mg/kg L-DOPA or saline, rats were placed into the center of a cage with a topunit equipped with light-emitting diodes and a charge-coupled device (CCD) camera (Phenotyper®, Noldus Information Technology, Wageningen, The Netherlands; open field dimensions: 45 × 45 × 56 cm). Durations (s) and frequencies (n) of motor and exploratory behaviors were rated blindly in blocks of 5 min for a total of 30 min using EthoVision XT (Noldus Information Technology, Wageningen, The Netherlands). Rated behaviors were: (A) ambulation as a measure of motor activity; (B) sitting as a measure of “passive immobility” according to Müller et al. (2004); (C) rearing (freely standing or leaning against the wall) as a measure of both motor activity and non-selective attention according to Aspide et al. (1998); (D) head-shoulder motility as a parameter of both motor activity and non-selective attention (Nikolaus et al., 2013, 2014b); (E) grooming (fur, paw and claw licking, scratching). In addition, based on the dislocation of the animal's center point, EthoVision XT automatically determined the distance in centimeters traveled by the rat. Following the behavioral trials, animals were anesthetized and administered [^123^I]IBZM as described above.

### Evaluation of D_2_ receptor imaging studies

Imaging data were evaluated using PMOD (version 3.5, PMOD Technologies Ltd., Zürich, Switzerland). Maximum striatal count rates (counts/pixel) were determined on coronal slices by defining a spherical region of interest (ROI; radius: 4 mm, volume: 0.16 cm^3^), which comprised a total of 6 pixels. On the same slices, cerebellar count rates (counts/pixel) were obtained by defining an ellipsoid reference region (REF; radius: 8 mm, volume: 0.35 cm^3^), which comprised a total of 13 pixels and was situated ~15 mm posterior to the frontal cortex corresponding anatomically to the rat cerebellum. Left and right striatal counts rates were averaged. The equilibrium ratios of the distribution volumes of the specifically and the non-specifically bound compartment [V${}_{3}^{\prime \prime }$ = V_T_(ROI)/V_T_(REF) − 1] were computed as estimates for the binding potentials (Laruelle et al., 1994).

### Statistical analysis

#### D_2_ receptor imaging studies

Distributions of binding data were tested for normality with the non-parametric Kolmogorov–Smirnov test (α = 0.05). In each pre-treatment condition (baseline, saline, benserazide, 5 and 10 mg/kg L-DOPA/benserazide), V${}_{3}^{\prime \prime }$-values were found to be normally distributed (0.09 ≤ *p* ≤ 0.20). Cerebellar count rates were not normally distributed in all pre-treatment conditions (0.0001 ≤ *p* ≤ 0.20). Therefore, striatal V${}_{3}^{\prime \prime }$-values and cerebellar radioactivity count rates were compared between pre-treatment conditions (baseline, benserazide, 5 and 10 mg/kg L-DOPA/benserazide) by paired *t*-test (two-tailed, α = 0.0167 after Bonferroni correction for multiple comparisons) and the Wilcoxon signed rank test for paired samples (two-tailed, α = 0.0167 after Bonferroni correction), respectively. In addition, striatal V${}_{3}^{\prime \prime }$-values and cerebellar radioactivity count rates were compared between treatment groups (5 and 10 mg/kg L-DOPA/benserazide, saline) with the *t*-test for independent samples (two-sided, α = 0.0167 after Bonferroni correction) and the Kruskal Wallis test for unrelated samples (two-tailed, α = 0.0167 after Bonferroni correction), respectively.

DA release is generally assessed *in vivo* by double measurement of D_2_ receptor binding—one time prior to and one time contingent on the administration of compounds, which lead to the increased availability of DA in the synaptic cleft (for review see Laruelle, 2000). The principle underlying this approach is the competition between endogenous DA and the administered D_2_ receptor radioligand The dissociation constant, K_D_, of the endogenous ligand DA is 7.5 nM (for review see Seeman and Grigoriadis, 1987), whereas for the exogenous ligand IBZM K_D_'s of 0.28 (Verhoeff et al., 1991) and 1.2 nM (de Paulis et al., 1988) were reported (mean K_D_, 0.74 nM). As the affinity, K, is defined as the reciprocal of the K_D_, the affinities of endogenous and exogenous ligand, K_endo_ and K_exo_, amount to 0.13 and 1.35 nM^−1^, respectively. In order to estimate the change of synaptic DA concentrations induced by the individual doses of L-DOPA, for each animal the percentual difference of [^123^I]IBZM binding relative to baseline was computed and multiplied with the ratio of affinities (K_exo_/K_endo_). Percentual reductions of radioligand binding relative to baseline (and estimated alterations of DA levels) were compared between 5 and 10 mg/kg L-DOPA/benserazide with the parametric independent *t*-test for unrelated samples (two-sided, α = 0.05).

Calculations were performed using IBM SPSS Statistics 22 (IBM SPSS Software Germany, Ehningen, Germany).

#### Behavioral studies

Distributions of behavioral data [traveled distance (cm), duration (s) and frequency (n) of ambulation, sitting, rearing, head-shoulder motility, and grooming in 5-min bins were assessed with the non-parametric Kolmogorov–Smirnov test (α = 0.05)]. Since the majority of behavioral parameters was not found to be normally distributed in any of the pre-treatment conditions (0.0001 ≤ *p* ≤ 0.2), behaviors in each 5-min time bin and over the whole trial (min 1–30) were compared between groups using the Mann–Whitney *U*-test for unrelated samples (two-sided, α = 0.0167 after Bonferroni correction). Calculations were performed using IBM SPSS Statistics 22.

The medians of the individual behavioral parameters obtained after saline (Y-axis: traveled distance, duration, and frequency of ambulation, sitting, rearing, head-shoulder motility, or grooming) were plotted against the end-points of the individual time frames (X-axis). Upon visual inspection of the data, the following mathematical models were fit to the individual behavioral parameters, using either linear or non-linear regression analysis with the regression coefficient (*R*^2^) as a measure for the goodness of fit: (1) *traveled distance:* exponential function [y(t) = a ^*^ exp (−K ^*^ x) + plateau with a, value at the time t; K, rate constant; t, time); *R*^2^ = 0.953; (2) *ambulation duration:* exponential function; *R*^2^ = 0.982; (3) *ambulation frequency:* exponential function; *R*^2^ = 0.964; (4) *sitting duration:* linear function (y = ax + b with a, slope and b, y-intercept); *R*^2^ = 0.920; (5) *sitting frequency:* quadratic function (y = a + bx + cx^2^ with a, absolute term; bx, linear term; cx^2^, quadratic term); *R*^2^ = 0.965; (6) *rearing duration:* exponential function; *R*^2^ = 0.877; (7) *rearing frequency:* exponential function; *R*^2^ = 0.933; (8) *duration of head-shoulder motility:* quadratic function; *R*^2^ = 0.966; (9*) frequency of head-shoulder motility:* linear function; *R*^2^ = 0.931).

The same models were fit to the behavioral data obtained after 5 and 10 mg/kg L-DOPA with *R*^2^-values, respectively, of 0.980 and 0.991 (traveled distance), 0.967 and 0.979 (ambulation duration), 0.966 and 0.997 (ambulation frequency), 0.945 and 0.958 (sitting duration), 0.682 and 0.767 (sitting frequency), 0.930 and 0.877 (rearing duration), 0.966 and 0.980 (rearing frequency), 0.979 and 0.958 (duration of head-shoulder motility), and 0.820 and 0.877 (frequency of head-shoulder motility). Due to the low expression of both grooming duration and grooming frequency, the grooming data could not be reasonably fit to any mathematical model. Curve fittings were performed using GraphPad Prism (version 3.0 for Windows, GraphPad Software, San Diego, USA). *t–b* Curves were compared between treatment groups using the *F*-test (α = 0.0167 after Bonferroni correction). In order to gauge the extent of association between D_2_ receptor binding and motor/exploratory parameters, Spearman rank correlation coefficients (*r*) were computed (α = 0.05) for V_3_″-values and behavioral data in min 1–5, 6–10, 11–15, 16–20, 21–25, 26–30, and 1–30. Calculations were performed using SigmaStat for Windows Version 3.5 (Systat Software Inc., Erkrath, Germany).

## Results

### D_2_ receptor imaging studies

**Figure 2:**
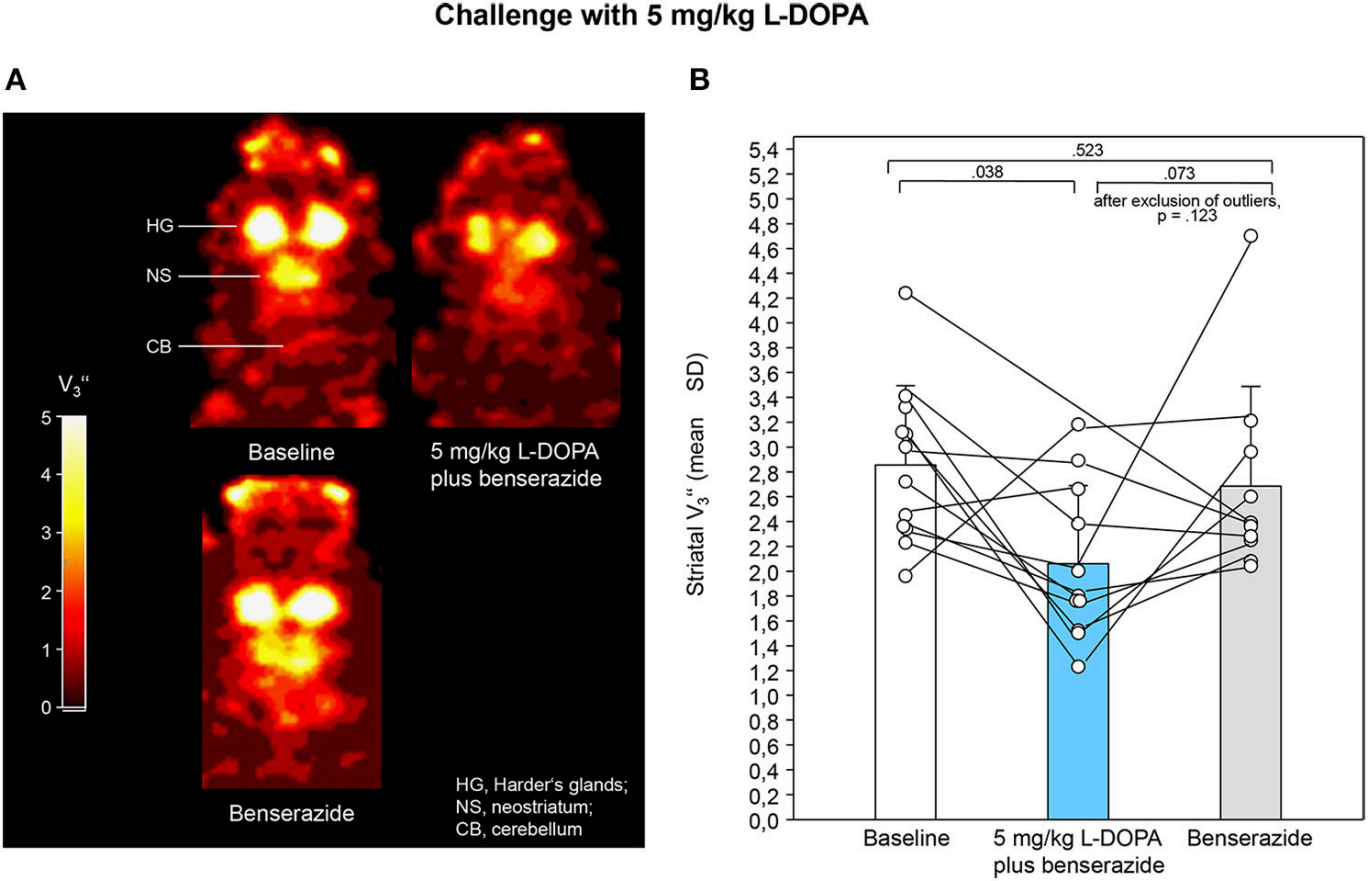
**(A)** Coronal [^123^I]IBZM images of the samt rat in baseline and after challenge with 5 mg/kg L-DOPA/benserazide and benserazide alone. All images show V${}_{3}^{\prime \prime }$-values; it is understood, that the calculation of V${}_{3}^{\prime \prime }$ is only valid for regions with specific radioligand binding such as the rat striatum. Calculations were performed using PMOD (version 3.5, PMOD Technologies Ltd., Zürich, Switzerland). **(B)** Striatal equilibrium ratios (V${}_{3}^{\prime \prime }$) in baseline and after challenge with 5 mg/kg L-DOPA/benserazide and benserazide alone. Rendered are means and standard deviations of the means. The circles represent the individual animals. For significant differences between conditions the respective *p*-values are given (two-tailed paired *t*-test, α = 0.0167 after Bonferroni correction). If the outlier in the benserazide condition (Figure 2B) was excluded, comparison of 5 mg/kg L-DOPA/benserazide and benserazide yielded a *p* of 0.123.

**Figure 3:**
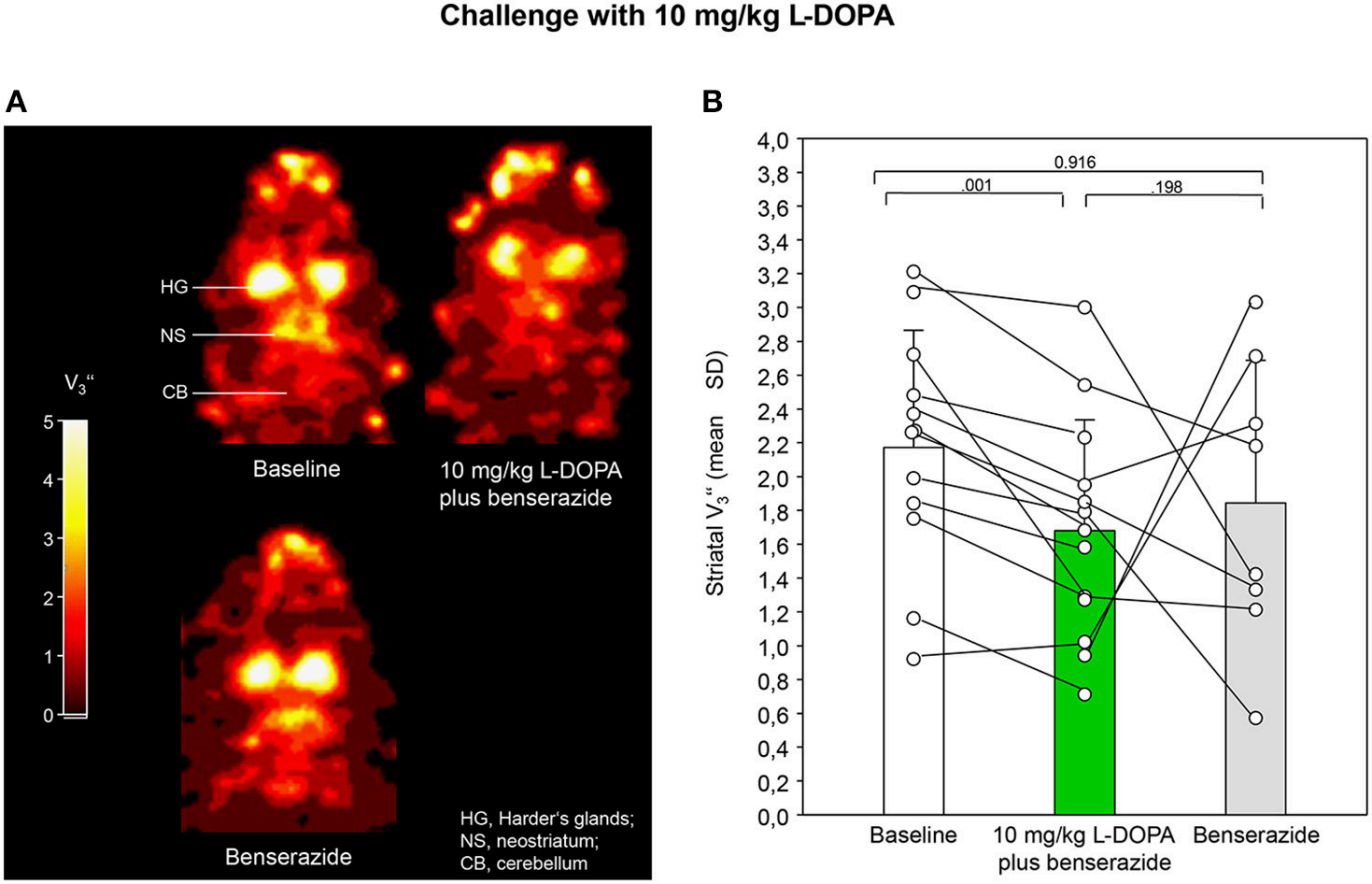
**(A)** Coronal [^123^I]IBZM images of the samt rat in baseline and after challenge with 10 mg/kg L-DOPA/benserazide and benserazide alone. All images show V${}_{3}^{\prime \prime }$-values; it is understood, that the calculation of V${}_{3}^{\prime \prime }$ is only valid for regions with specific radioligand binding such as the rat striatum. Calculations were performed using PMOD (version 3.5, PMOD Technologies Ltd., Zürich, Switzerland). **(B)** Striatal equilibrium ratios (V${}_{3}^{\prime \prime }$) in baseline and after challenge with 10 mg/kg L-DOPA/benserazide and benserazide alone. Rendered are means and standard deviations of the means. The circles represent the individual animals. For significant differences between conditions the respective *p*-values are given (two-tailed paired *t*-test, α = 0.0167 after Bonferroni correction).

**Figure 4:**
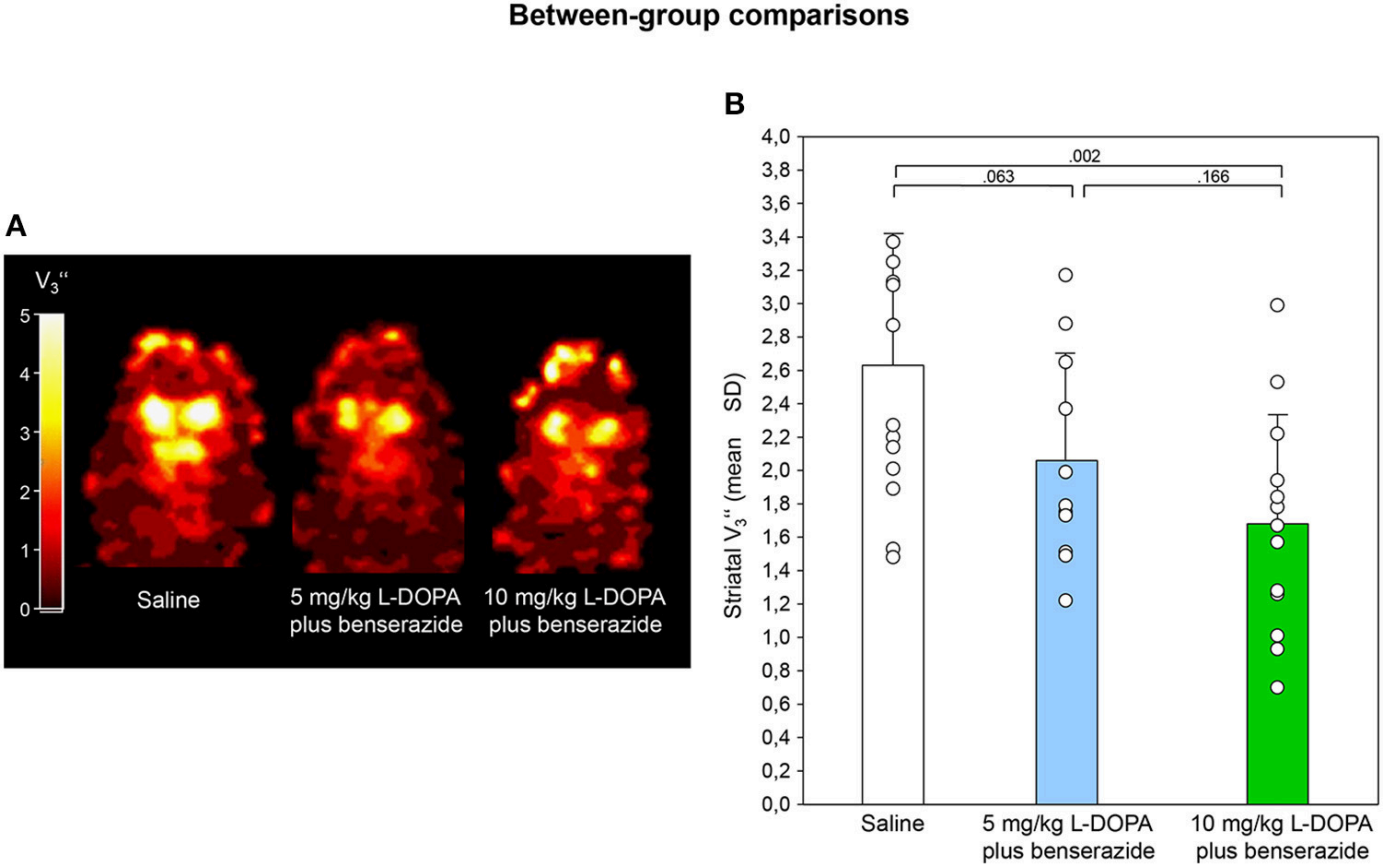
**(A)** Coronal [^123^I]IBZM images of different rats after challenge with saline, 5 and 10 mg/kg L-DOPA/benserazide. All images show V${}_{3}^{\prime \prime }$ values; it is understood, that the calculation of $V_{3}^{\prime \prime }$ is only valid for regions with specific radioligand binding such as the rat striatum. Calculations were performed using PMOD (version 3.5, PMOD Technologies Ltd., Zürich, Switzerland). **(B)** Striatal equilibrium ratios (V${}_{3}^{\prime \prime }$) after challenge with saline, 5 and 10 mg/kg L-DOPA/benserazide. Rendered are means and standard deviations of the means. The circles represent the individual animals. For significant between-group differences the respective *p*-values are given (two-tailed independent *t*-test, α = 0.0167 after Bonferroni correction).

Figures 2A, 3A show characteristic images of [^123^I]IBZM accumulation on coronal slices in baseline and after challenge with 5 and 10 mg/kg L-DOPA/benserazide and benserazide alone. Striatal [^123^I]IBZM accumulations are markedly reduced following pre-treatment with both 5 mg/kg (Figure 2A) and 10 mg/kg L-DOPA/benserazide (Figure 3A). SPECT images were obtained on the same rats. Figure 4A shows characteristic images of [^123^I]IBZM accumulations on coronal slices of different rats after treatment with saline and 5 and 10 mg/kg L-DOPA/benserazide and 10 mg/kg L-DOPA/benserazide. Again, striatal [^123^I]IBZM accumulations were lower following challenge with both 5 and 10 mg/kg L-DOPA/benserazide.

After application of 5 mg/kg (Figure 2B) and 10 mg/kg L-DOPA/benserazide (Figure 3B), striatal V${}_{3}^{\prime \prime }$-values were 2.06 ± 0.63 (mean ± SD) and 1.68 ± 0.66, respectively. Baseline V${}_{3}^{\prime \prime }$ amounted to 2.86 ± 0.64 (5 mg/kg L-DOPA/benserazide) and 2.17 ± 0.69 (10 mg/kg L-DOPA/benserazide), while, after benserazide, V${}_{3}^{\prime \prime }$-values of 2.69 ± 0.63 (5 mg/kg L-DOPA/benserazide) and 1.85 ± 0.84 (10 mg/kg L-DOPA/benserazide) were obtained. The comparison of baseline and 5 mg/kg L-DOPA/benserazide yielded no significant difference after Bonferroni correction (*p* = 0.038). Likewise, differences between baseline and benserazide (*p* = 0.523) and between 5 mg/kg L-DOPA/benserazide and benserazide (*p* = 0.073) were not significant. Comparison between baseline and 10 mg/kg L-DOPA/benserazide yielded a *p* = 0.001. The differences between baseline and benserazide (*p* = 0.916) and between 10 mg/kg L-DOPA/benserazide and benserazide (*p* = 0.198) were not significant.

After saline, striatal V${}_{3}^{\prime \prime }$ was 2.63 ± 0.78 (Figure 4B). Comparisons between saline and 5 mg/kg L-DOPA/benserazide, between saline and 10 mg/kg L-DOPA/benserazide as well as between 5 and 10 mg/kg L-DOPA/benserazide yielded *p*-values of 0.063, 0.002, and 0.166, respectively.

Alterations of D_2_ receptor binding relative to baseline amounted to 79 ± 35% after 5 mg/kg L-DOPA/benserazide and to 81 ± 15% after 10 mg/kg L-DOPA/benserazide. Multiplication of percentual decreases by K_exo_/K_endo_ yielded mean increases of synaptic DA by 827 ± 361% after 5 mg/kg L-DOPA/benserazide and by 840 ± 161% after 10 mg/kg L-DOPA/benserazide. Comparison between groups yielded a *p* = 0.907.

After 5 and 10 mg/kg L-DOPA/benserazide, median cerebellar radioactivity concentrations amounted to 585 counts/pixel (25-percentile: 512 counts/pixel, 75-percentile: 689 counts/pixel) and 689 counts/pixel (25-percentile: 557 counts/pixel, 75-percentile: 798 counts/pixel), respectively. In baseline, median cerebellar count rates were 551 counts/pixel (25-percentile: 512 counts/pixel, 75-percentile: 624 counts/pixel) and 532 counts/pixel (25-percentile: 512 counts/pixel, 75-percentile: 853 counts/pixel), while after benserazide, cerebellar radioactivity concentrations amounted to 512 counts/pixel (25-percentile: 473 counts/pixel, 75-percentile: 591 counts/pixel) and 768 counts/pixel (25-percentile: 532 counts/pixel, 75-percentile: 811 counts/pixel) in the 5 and 10 mg/kg L-DOPA/benserazide condition, respectively. No significant differences were found between treatment conditions (0.123 ≤ *p* ≤ 0.859).

After saline, median cerebellar radioactivity concentrations were 627 counts/pixel (25-percentile: 527 counts/pixel, 75-percentile: 694 counts/pixel). No significant between-group differences were obtained (*p* = 0.255).

### Behavioral studies

#### Median differences and *t–b* curves–traveled distance

**Figure 5:**
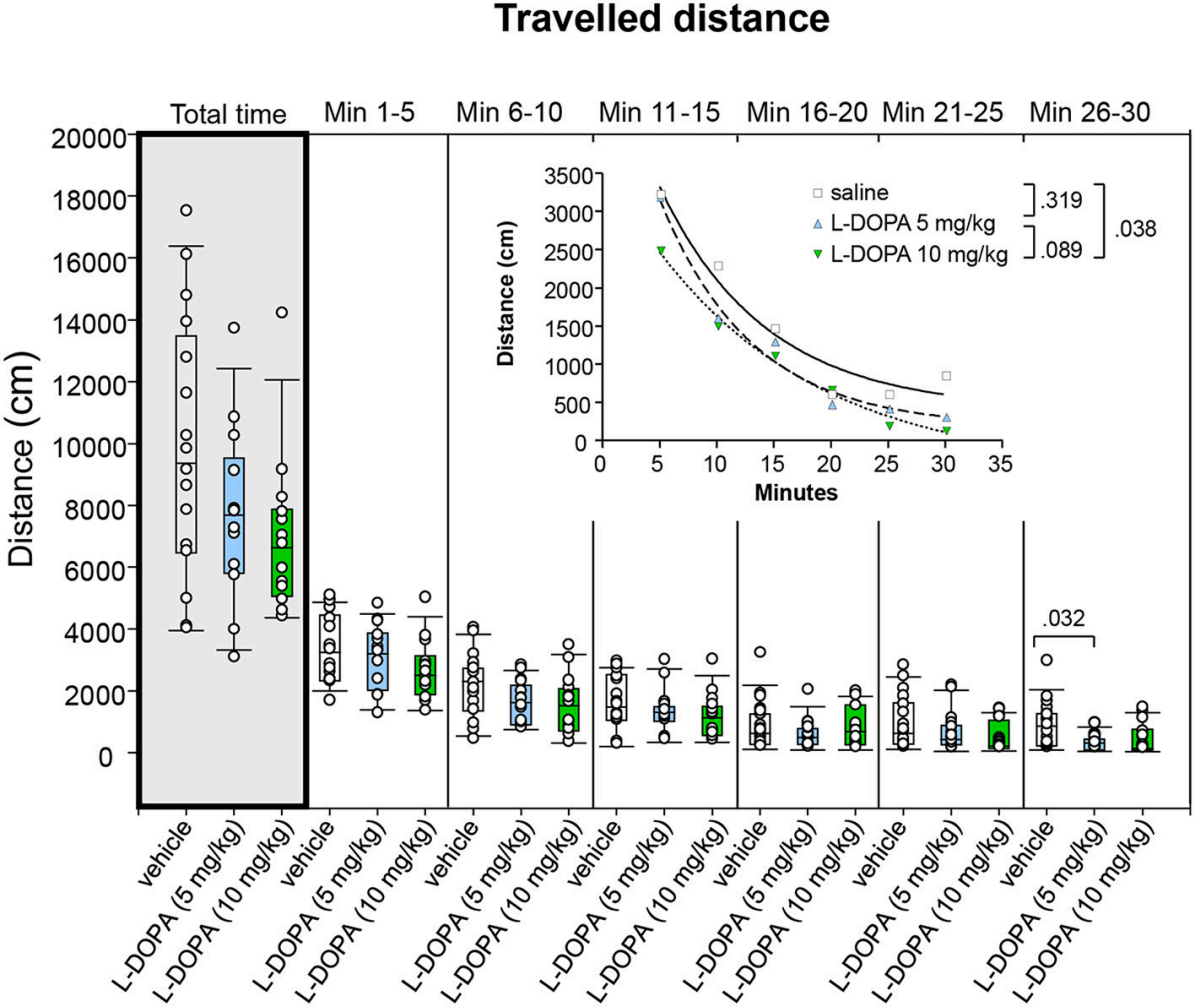
**Traveled distance (cm) after vehicle (0.9% saline), 5 and 10 mg/kg L-DOPA**. The figure shows box and whisker plots of median distances traveled during the whole time of testing (gray shade) and in the individual 5-min time bins. 25-/75-percentiles are given in the boxes, while 5-/95-percentiles are represented by the whiskers. The circles represent the individual animals. For significant between-group differences the respective *p*-values are given (two-tailed Mann–Whitney *U*-test, α = 0.0167 after Bonferroni correction). Inset: *t–b* Curves obtained by plotting median values of traveled distances against time and fitting exponential functions [y(t) = a ^*^ exp (−K ^*^ x) + plateau with a, value at the time t; -K, rate constant; t, time] to these data. For the comparisons between groups (two-tailed *F*-test, α = 0.0167 after Bonferroni correction) the respective *p*-values are given).

Comparison of traveled distance between animals treated with 10 mg/kg L-DOPA/benserazide and saline and between animals treated with 5 and 10 mg/kg L-DOPA/benserazide yielded no significant difference in any of the time bins (0.062 ≤ *p* ≤ 1.0; Figure 5). Also the median traveled distance in min 26–30 after 5 mg/kg L-DOPA/benserazide was not significantly different from saline (*p* = 0.032) after Bonferroni correction.

*t–b* Curves neither differed significantly between 5 mg/kg L-DOPA/benserazide and saline (*p* = 0.319) nor between 5 and 10 mg/kg L-DOPA/benserazide (*p* = 0.089). Comparison of *t–b* curves after 10 mg/kg L-DOPA/benserazide (a, 4065 ± 232; K, 0.069 ± 0.017; plateau, −403.9±341.5) and saline (a, 5016 ± 929; K, 0.108 ± 0.045; plateau, 402.9 ± 439) yielded no significant difference after Bonferroni correction (*p* = 0.038). Nevertheless, both curve plateau and rate constant after 10 mg/kg L-DOPA/benserazide were lower compared to saline. This suggests that after the higher L-DOPA dose, the traveled distance decreased at a *slower* rate to a *lower* final level relative to control.

#### Ambulation

**Figure 6:**
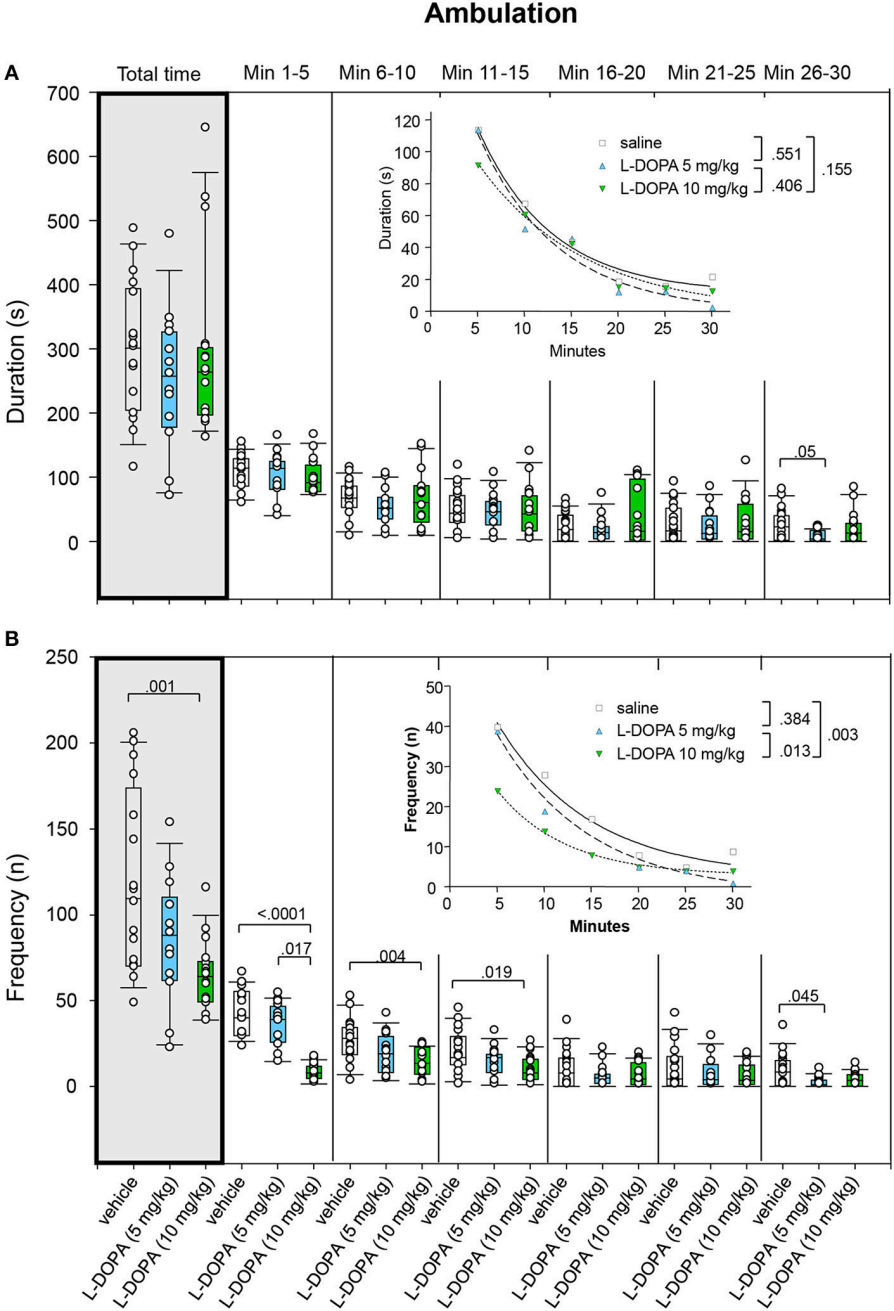
**Ambulation. (A)** Duration (s) and **(B)** frequency (n) after vehicle (0.9% saline), 5 and 10 mg/kg L-DOPA. The figure shows box and whisker plots of median ambulation durations and frequencies during the whole time of testing (gray shade) and in the individual 5-min time bins. 25-/75-percentiles are given in the boxes, while 5-/95-percentiles are represented by the whiskers. The circles represent the individual animals. For significant between-group differences the respective *p*-values are given (two-tailed Mann–Whitney *U*-test, α = 0.0167 after Bonferroni correction). Insets: *t–b* Curves obtained by plotting median values of ambulation durations **(A)** and ambulation frequencies **(B)** against time and fitting exponential functions [y(t) = a ^*^ exp (−K ^*^ x) + plateau with a, value at the time t; -K, rate constant; t, time] to these data. For the comparisons between treatment groups (two-tailed *F*-test, α = 0.0167 after Bonferroni correction) the respective *p*-values are given.

Ambulation duration neither differed signficantly between animals treated with 10 mg/kg L-DOPA/benserazide and saline nor between animals treated with 5 and 10 mg/kg L-DOPA/benserazide in any of the time bins (0.220 ≤ *p* ≤ 0.9432; Figure 6A). Likewise, after Bonferroni correction, the median duration of ambulation in min 26–30 after 5 mg/kg L-DOPA/benserazide was not significantly different from the median duration of ambulation after saline (*p* = 0.05).

Comparison of *t–b* curves yielded no significant difference (0.155 ≤ *p* ≤ 0.551).

Rats treated with 5 mg/kg L-DOPA/benserazide displayed a significantly lower ambulation frequency (Figure 6B) compared to saline in min 1–5 (*p* = 0.017). Comparison of ambulation frequency after 5 mg/kg L-DOPA/benserazide and saline in min 26–30 yielded no significant difference after Bonferroni correction (*p* = 0.045). After 10 mg/kg L-DOPA/benserazide, animals moved significantly less frequently compared to saline in min 1–5 and 6–10 as well as during the whole testing time (0.0001 ≤ *p* ≤ 0.004). There were no differences between 10 mg/kg L-DOPA/benserazide and saline in min 11–15 after Bonferroni correction (*p* = 0.019). Significant between-group differences were evident starting with the first time frame.

*t–b* Curves differed significantly between 10 mg/kg L-DOPA/benserazide (exponential fit; a, 42.51 ± 2.38; K, 0.14 ± 0.01; plateau, 2.82 ± 0.56) and saline (a, 63.96 ± 9.68; K, 0.10 ± 0.04; plateau, 2.61 ± 5.34; *p* = 0.003) as well as between 10 and 5 mg/kg L-DOPA/benserazide (a, 66.49 ± 9.73; K, 0.10 ± 0.04; plateau, 6.79 ± 5.39; *p* = 0.013). After 10 mg/kg L-DOPA/benserazide, the rate constant was lower compared to saline. This suggests that after 10 mg/kg L-DOPA/benserazide, ambulatory frequency decreased at a *faster* rate. After 10 mg/kg L-DOPA/benserazide, however, both rate constant and plateau were higher compared to 5 mg/kg L-DOPA/benserazide indicating a *faster* rate of increase to a *higher* final level.

#### Sitting

**Figure 7:**
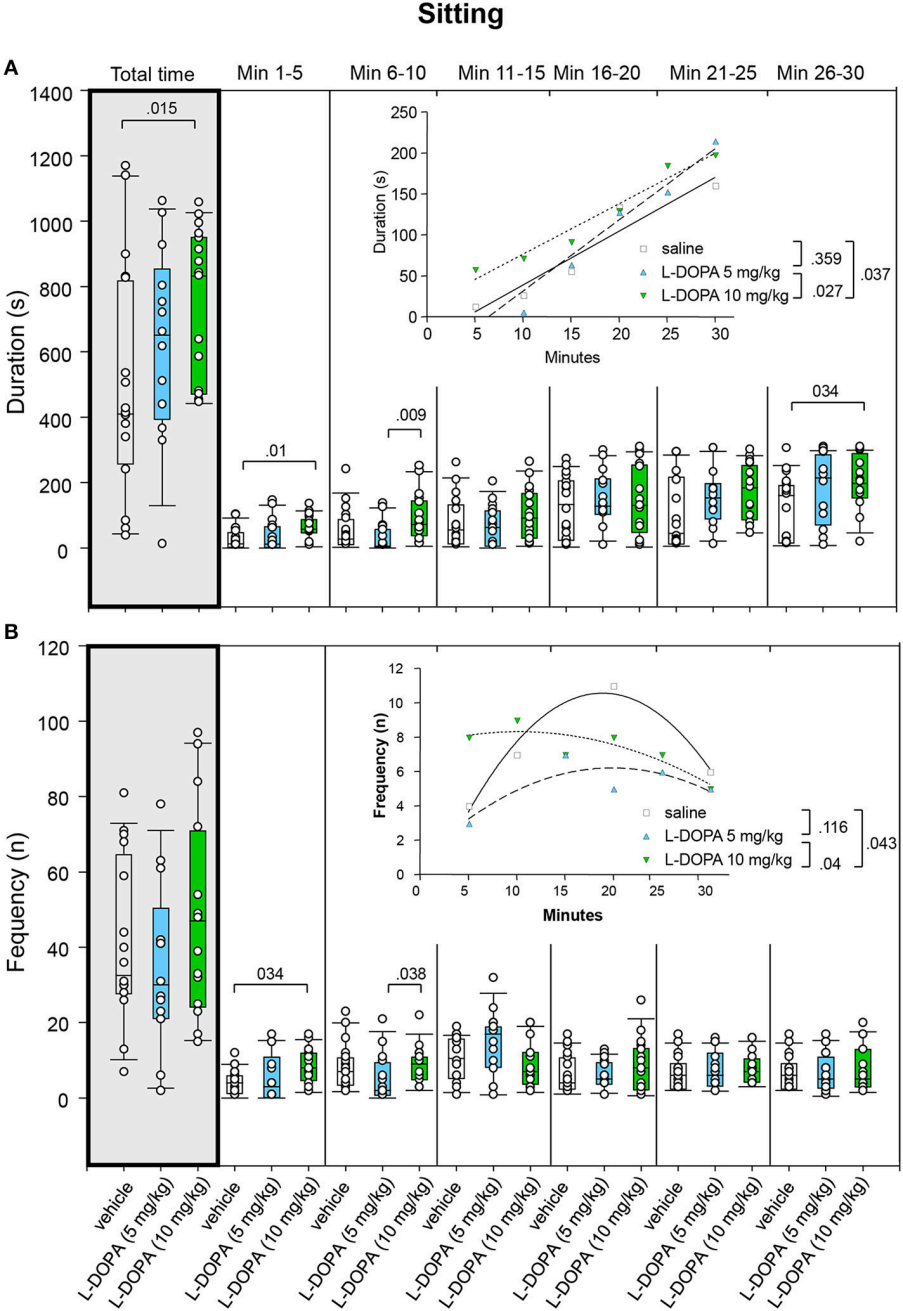
**Sitting. (A)** Duration (s) and **(B)** frequency (n) after vehicle (0.9% saline), 5 and 10 mg/kg L-DOPA. The figure shows box and whisker plots of median sitting durations and frequencies during the whole time of testing (gray shade) and in the individual 5-min time bins. 25-/75-percentiles are given in the boxes, while 5-/95-percentiles are represented by the whiskers. The circles represent the individual animals. For significant between-group differences the respective *p*-values are given (two-tailed Mann–Whitney *U*-test, α = 0.0167 after Bonferroni correction). Insets: *t–b* Curves obtained by plotting median values of sitting durations **(A)** and sitting frequencies **(B)** against time. Linear functions (y = ax + b with a, slope and b, y-intercept) were fitted to the plots of sitting durations, whereas quadratic functions (y = a + bx + cx^2^ with a, absolute term; bx, linear term; cx^2^, quadratic term) were fitted to the plots of sitting frequencies. For the comparisons between treatment groups (two-tailed *F*-test, α = 0.0167 after Bonferroni correction) the respective *p*-values are given.

After 5 mg/kg L-DOPA/benserazide, animals displayed significantly longer sitting behavior (Figure 7A) in min 6–10 (*p* = 0.009) compared to rats treated with saline. After 10 mg/kg L-DOPA/benserazide, rats sat for a significantly longer time than in the saline condition in min 1–5 (*p* = 0.010) as well as throughout the whole trial (*p* = 0.015). Comparison between 10 mg/kg L-DOPA/benserazide and saline in min 25–30 yielded no significant difference after Bonferroni correction (*p* = 0.034). Significant between-group differences were evident from the first time frame, but temporarily disappeared during the third to fifth time frame (min 11–25).

*t–b* Curves did not differ between 5 mg/kg L-DOPA/benserazide and saline (*p* = 0.359). Likewise, comparison of *t–b* curves after 10 mg/kg L-DOPA/benserazide (linear fit; slope, 6.15 ± 0.64; y-intercept, 14.80 ± 12.6) and saline (slope, 6.57 ± 1.12; y-intercept, −26.84 ± 20.32; *p* = 0.037) as well as after 10 and 5 mg/kg L-DOPA/benserazide (slope, 8.69 ± 0.96; y-intercept, −55.67 ± 18.66; *p* = 0.027) yielded no significant difference after Bonferroni correction. Nevertheless, the slope of the 5 mg/kg curve exceeded the slopes of the saline and the 10 mg/kg curve, indicating a *faster* rate of increase of sitting duration to a *higher* final level after the lower L-DOPA dose.

Comparison of sitting frequency (Figure 7B) between 10 mg/kg L-DOPA/benserazide and saline in min 1–5 (*p* = 0.034) and between 5 and 10 mg/kg L-DOPA/benserazide in min 6–10 (*p* = 0.038) yielded no significant difference after Bonferroni correction. Moreover, no significant difference was found between 5 mg/kg L-DOPA/benserazide and saline (0.083 ≤ *p* ≤ 0.948).

Comparisons of *t–b* curves after 10 mg/kg L-DOPA/benserazide (quadratic fit; a, 7.50 ± 1.52; b, 0.16 ± 0.20; c, −0.01 ± 0.006) and saline (a, −2.28 ± 1.84; b, 1.36 ± 0.26; c, −0.04 ± 0.007; *p* = 0.04) as well as after 5 (a, 0.87 ± 2.16; b, 0.54 ± 0.28; c, −0.01 ± 0.01; *p* = 0.04) and 10 mg/kg L-DOPA/benserazide yielded no significant difference after Bonferroni correction. Still, after 5 and 10 mg/kg L-DOPA/benserazide, the linear term was lower and the quadratic term was higher compared to saline suggesting a *slower* rate of increase of sitting frequency to a *higher* final level.

#### Rearing

**Figure 8:**
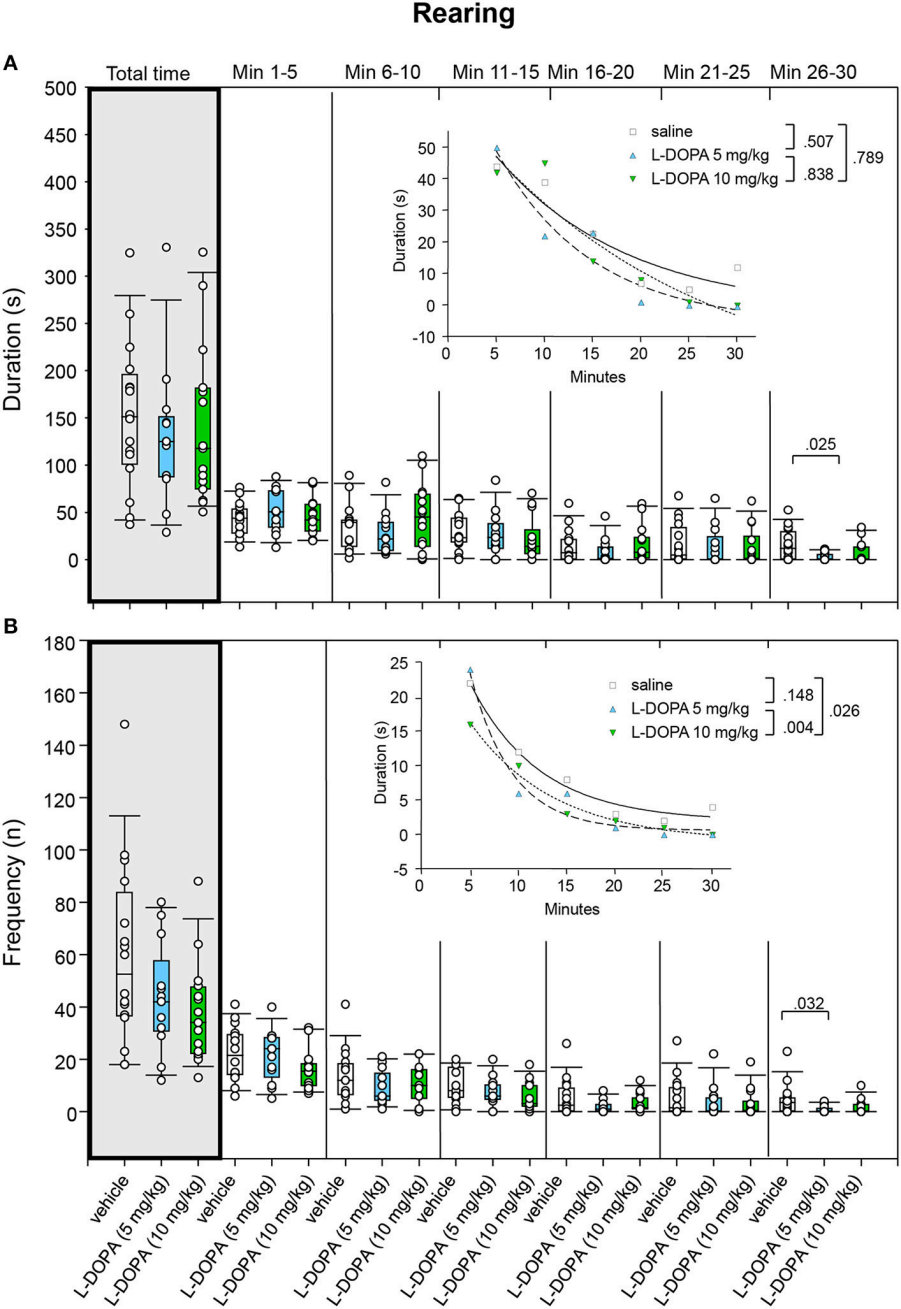
**Rearing**. **(A)** Duration (s) and **(B)** frequency (n) after vehicle (0.9% saline), 5 and 10 mg/kg L-DOPA. The figure shows box and whisker plots of median rearing durations and frequencies during the whole time of testing (gray shade) and in the individual 5-min time bins. 25-/75-percentiles are given in the boxes, while 5-/95-percentiles are represented by the whiskers. The circles represent the individual animals. For significant between-group differences the respective *p*-values are given (two-tailed Mann–Whitney *U*-test, α = 0.0167 after Bonferroni correction). Insets: *t–b* Curves obtained by plotting median values of rearing durations **(A)** and rearing frequencies **(B)** against time and fitting exponential functions [y(t) = a ^*^ exp (−K ^*^ x) + plateau with a, value at the time t; -K, rate constant; t, time] to these data. For the comparisons between treatment groups (two-tailed *F*-test, α = 0.0167 after Bonferroni correction) the respective *p*-values are given.

Duration of rearing neither differed signficantly between animals treated with 10 mg/kg L-DOPA/benserazide and saline nor between animals treated with 5 and 10 mg/kg L-DOPA/benserazide in any of the time bins (0.142 ≤ *p* ≤ 1.0; Figure 8A). Moreover, comparison of rearing duration between 5 mg/kg L-DOPA/benserazide and saline in min 26–30 yielded a *p*-value (*p* = 0.025), which was not significant after Bonferroni correction.

Likewise, comparison of *t–b* curves yielded no significant differences (0.127 ≤ *p* ≤ 0.752).

Rearing frequency did not differ significantly between animals treated with 10 mg/kg L-DOPA/benserazide and saline and between animals treated with 5 and 10 mg/kg L-DOPA/benserazide (0.058 ≤ *p* ≤ 0.843; Figure 8B). Furthermore, comparison of rearing frequency between 5 mg/kg L-DOPA/benserazide and saline in min 26–30 yielded no significant difference after Bonferroni correction (*p* = 0.032).

*t–b* Curves differed significantly between 5 (exponential fit; a, 91.11 ± 19.37; K, 0.10 ± 0.05; plateau, −5.95 ± 11.02) and 10 mg/kg L-DOPA benserazide (a, 95.45 ± 54.29; K, 0.04 ± 0.06; plateau, −30.37 ± 70.11; *p* = 0.004). The higher rate constant after 5 mg/kg L-DOPA/benserazide suggested a *higher* rate of decrease of rearing frequency, while the higher plateau value indicated a *higher* final level of rearing frequency compared to the higher L-DOPA dose. The difference of *t–b* curves after 10 mg/kg L-DOPA and saline (a, 71.03 ± 15.36; K, 0.073 ± 0.067; plateau, −2.11 ± 21.08; *p* = 0.026) was not significant after Bonferroni correction. Yet, the lower rate constant after 10 mg/kg L-DOPA/benserazide implied a (slightly) *lower* rate of decrease of rearing frequency, while the lower plateau value indicated a *lower* final level of rearing frequency compared to saline.

#### Head-shoulder motility

**Figure 9:**
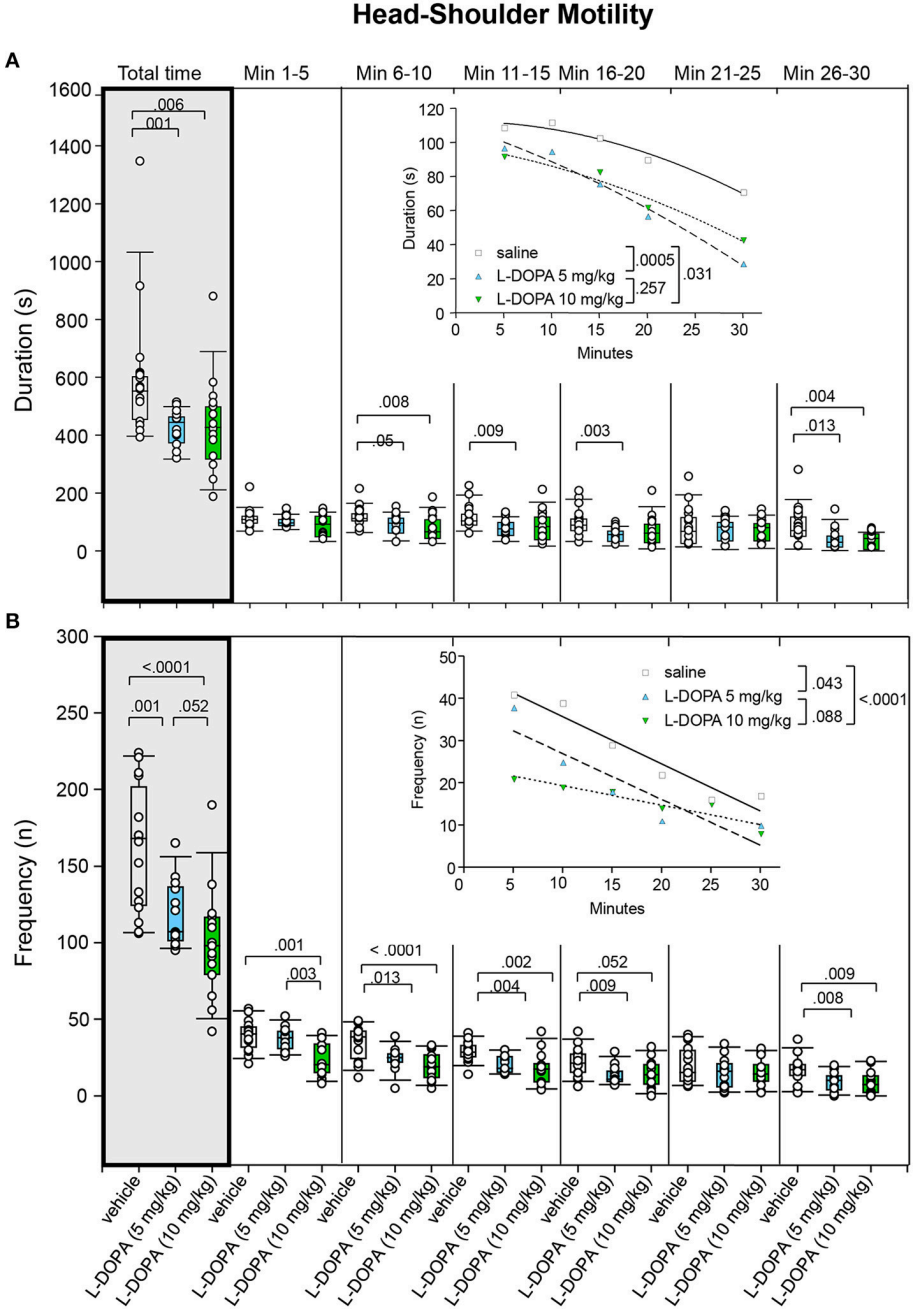
**Head-shoulder motility**. **(A)** Duration (s) and **(B)** frequency (n) after vehicle (0.9% saline), 5 and 10 mg/kg L-DOPA. The figure shows box and whisker plots of median durations and frequencies of head-shoulder motility during the whole time of testing (gray shade) and in the individual 5-min time bins. 5-/75-percentiles are given in the boxes, while 5-/95-percentiles are represented by the whiskers. The circles represent the individual animals. For significant between-group differences the respective *p*-values are given (two-tailed Mann–Whitney *U*-test, α = 0.0167 after Bonferroni correction). Insets: *t–b* Curves obtained by plotting median values of motility durations **(A)** and frequencies **(B)** against time. Quadratic functions (y = a + bx + cx^2^ with a, absolute term; bx, linear term; cx^2^, quadratic term) were fitted to the plots of motility durations, while linear functions (y = ax + b with a, slope and b, y-intercept) were fitted to the plots of motility frequencies. For the comparisons between grousp (two-tailed *F*-test, α = 0.0167 after Bonferroni correction) the respective *p*-values are given.

After treatment with 5 mg/kg L-DOPA/benserazide, the duration of head-shoulder motility (Figure 9A) was significantly shorter relative to saline in min 11–15 (*p* = 0.009), 16–20 (*p* = 0.003), and 26–30 (*p* = 0.013) as well as throughout the whole trial (*p* = 0.001). However, there was no significant difference in min 6–10 after Bonferroni correction (*p* = 0.05). Head-shoulder motility was significantly shorter after 10 mg/kg L-DOPA/benserazide relative to saline in min 6–10 and 26–30 as well as throughout the whole trial (0.004 ≤ *p* ≤ 0.008). Significant between-group differences were evident from min 6.

*t–b* Curves differed significantly between 5 mg/kg L-DOPA/benserazide (quadratic fit; a, 110.30 ± 10.74; b, −1.85 ± 1.41; c; −0.030 ± 0.039) and saline (a, 112.50 ± 8.00; b, 0.02 ± 1.05; c; −0.05 ± 0.03; *p* = 0.0005). However, no significant difference was obtained between *t–b* curves after 10 mg/kg L-DOPA/benserazide (a, 98.31 ± 14.41; b, −0.88 ± 1.86; c; −0.03 ± 0.05; *p* = 0.031) and saline after Bonferroni correction. Moreover, there was no difference between 5 and 10 mg/kg L-DOPA/benserazide (*p* = 0.257). Notably, after both L-DOPA doses, linear and quadratic terms were lower relative to saline suggesting a *slower* rate of decrease from a *lower* maximum level after L-DOPA.

Rats treated with 5 mg/kg L-DOPA/benserazide moved their head and shoulders (Figure 9B) significantly less frequently in min 6–11, 11–15, 16–20, and 26–30 as well as throughout the whole trial (0.001 ≤ *p* ≤ 0.013). After treatment with 10 mg/kg L-DOPA/benserazide, the median frequency of head-shoulder motility was decreased relative to saline in min 1–5, 6–10, 11–15, and 26–30 as well as during the whole testing time (0.0001 ≤ *p* ≤ 0.009). The frequency of head-shoulder motility was significantly elevated after 5 relative to 10 mg/kg L-DOPA/benserazide in min 1–6 (*p* = 0.003). Comparisons between 10 mg/kg L-DOPA/benserazide and saline in min 16–20 as well as between 5 and 10 mg/kg L-DOPA/benserazide in min 1–30 yieled no significant difference after Bonferroni correction (*p* = 0.052, each). Significant between-group differences were evident from the first time frame on, but temporarily disappeared during the fifth time frame (min 21–25).

*t–b* Curves differed significantly between 10 mg/kg L-DOPA/benserazide (linear fit; slope, −0.46 ± 0.09; y-intercept, 23.93 ± 1.69) and saline (slope, −1.12 ± 0.15; y-intercept, 46.93 ± 2.96; *p* < 0.0001). No difference was obtained between 5 and 10 mg/kg L-DOPA/benserazide (*p* = 0.088). Likewise, curves after 5 mg/kg L-DOPA/benserazide (slope, −1.09 ± 0.03; y-intercept, 37.78 ± 5.35) and saline were not different after Bonferroni correction (*p* = 0.043). Slopes as well as y-intercepts were lower after both L-DOPA doses relative to saline, indicating that the frequency of head and shoulder motility decreased at a *slower* rate from a *lower* maximum level.

#### Grooming

Comparison of grooming duration between animals treated with 10 mg/kg L-DOPA/benserazide and saline yielded no significant difference in any of the time bins (0.131 ≤ *p* ≤ 0.984; data not shown). Likewise, after Bonferroni correction, grooming duration did not differ between 5 mg/kg L-DOPA/benserazide and saline during the whole trial (*p* = 0.04). Yet, rats groomed for a significantly longer time after 5 mg/kg L-DOPA/benserazide relative to saline in min 6–10 (*p* < 0.0001). Moreover, grooming duration was longer in animals treated with 5 mg/kg compared to animals treated with 10 mg/kg L-DOPA/benserazide in min 6–10 (*p* ≤ 0.0001) as well as throughout the whole trial (*p* = 0.001).

Comparison of grooming frequency between 5 mg/kg L-DOPA/benserazide and saline as well as between 10 mg/kg L-DOPA/benserazide and saline yielded no significant difference in any of the time bins (0.085 ≤ *p* ≤ 0.918; data not shown). After 5 mg/kg L-DOPA/benserazide, rats groomed with a significantly higher frequency relative to 10 mg/kg L-DOPA/benserazide in min 6–10 (*p* = 0.007) as well as throughout the whole testing time (*p* = 0.001).

### Correlation of motor/exploratory behaviors with D_2_ receptor binding

After 5 mg/kg L-DOPA/benserazide, D_2_ receptor binding correlated negatively with sitting frequency in min 21–25 (*r* = – 0.596, *p* = 0.047). A marginal positive correlation was obtained between D_2_ receptor binding and rearing frequency in min 26–30 (*r* = 0.547, *p* = 0.076).

After 10 mg/kg L-DOPA/benserazide, D_2_ receptor binding correlated positively with sitting duration in min 25–30 (*r* =0.714, *p* = 0.037), with the frequency of head-shoulder motility in min 1–30 (*r* = 0.683, *p* = 0.036), and with grooming frequency in min 11–15 (*r* = 0.795, *p* = 0.01) and min 1–30 (*r* = 0.647, *p* = 0.030). Marginal positive correlations were obtained between D_2_ receptor binding and rearing frequency in min 1–5 (*r* = 0.635, *p* = 0.072) and the duration of head-shoulder motility in min 6–10 (*r* = 0.635, *p* = 0.072), while a marginal negative correlation was found between D_2_ receptor binding and rearing frequency in min 26–30 (*r* = 0.635, *p* = 0.059).

No significant correlations with behavioral parameters were observed after saline (0.231 ≤ *p* ≤ 0.976).

## Discussion

### D_2_ receptor binding

Challenge with both 5 and 10 mg/kg L-DOPA/benserazide reduced D_2_ receptor binding relative to baseline and saline. In each case, however, only the high dose challenge had a significant effect after Bonferroni correction. Cerebellar radioactivity concentrations were not significantly influenced by L-DOPA challenges indicating that no confounding effects were exerted on radioligand accumulation, e.g., by affecting cerebral perfusion. D_2_ receptor binding also did not differ between baseline and benserazide challenge and between L-DOPA/benserazide and treatment with benserazide alone, showing that the effects of L-DOPA on D_2_ receptor binding were not confounded by the joint administration of the AADC inhibitor.

We admistered L-DOPA 30 min prior to [^123^I]IBZM, and SPECT measurements were performed from 75 to 135 min post-challenge. Since maximum striatal DA concentrations are reached 40 min after i.p. L-DOPA and remain stable for ~2 h (e.g., de Souza Silva et al., 1997), our D_2_ receptor binding data were acquired during the maximum DAergic response. Changes in of synaptic DA concentrations were estimated by multiplying the percentual alteration of exogenous ligand binding with the ratio of affinities (K_exo_/K_endo_), resulting in mean increases of DA by 827 ± 361% after 5 mg/kg and to 840 ± 161% after 10 mg/kg L-DOPA/benserazide. This order of magnitude is in agreement with the increase of endogenous DA to 317% of baseline levels observed 60 min after 50 mg/kg L-DOPA/benserazide in an *in vivo* microdialysis study (de Souza Silva et al., 1997). The increases in synaptic DA did not differ significantly between the 5 and 10 mg/kg doses of L-DOPA/benserazide, indicating that the 5 mg/kg L-DOPA dose was sufficient to elicit maximum DA release into the synaptic cleft.

Our results on D_2_ receptor binding are consistent with the *in vivo* findings of Sossi et al. (2009) and Sahin et al. (2014), who observed reductions of [^11^C]raclopride and [^18^F]fallypride binding, respectively, relative to baseline in the degenerated striatum of 6-OHDA lesioned rats. However, they are not congruent with the results of Opacka-Juffry et al. (1998), who observed an increased D_2_ receptor binding relative to untreated controls in healthy rats. Moreover, they contradict the findings of Hume et al. (1995), who reported an elevation of D2 receptor binding in the lesioned striatum relative to the intact contralateral side and to unlesioned rats and an increase in the unlesioned striatum relative to healthy controls.

There is evidence that anesthetics may influence extracellular DA levels (for review see Müller et al., 2011). Both ketamine—the anesthetic used in the present investigation—and isoflurane—the anesthetic employed by Hume et al. (1995), Opacka-Juffry et al. (1998), Sossi et al. (2009), and Sahin et al. (2014)—have been shown to enhance DA efflux (for review see Müller et al., 2011). Thus, it may no be excluded that ketamine and isoflurance contributed to the reduction of D_2_ receptor binding observed in the present investigation as well as by Sossi et al. (2009) and Sahin et al. (2014). Since Hume et al. (1995) and Opacka-Juffry et al. (1998) also used isoflurance anesthesia, it may be ruled out, however, that the discrepancy of findings was solely due to anesthesia effects.

Yet, experimental procedures were widely discrepant: we injected L-DOPA 30 min prior to [^123^I]IBZM administration and acquired static SPECT images from min 75 to min 135 post-challenge; Sahin et al. (2014) also applied L-DOPA 30 min prior to [^16^F]fallypride but perfomed dynamic PET from min 30 to min 120 post-challenge; and Sossi et al. (2009) administerd L-DOPA 44 min prior to [^11^C]raclopride and conducted dynamic PET from min 45 to min 95 post-challenge, while Hume et al. (1995) as well as Opacka-Juffry et al. (1998) administerd L-DOPA 90 and 100 min, respectively, prior to [^11^C]raclopride and conducted dynamic PET from min 90 to min 160, post-challenge. Thus, the studies not only differed in the employed radioligands and the methods of data acquisition, but—more importantly—also in the time windows post-challenge, during which D_2_ receptor imaging data were acquired.

*In vivo* microdialysis studies have demonstrated that DA molecules—practically immediately upon injection of L-DOPA—start to accumulate in the synaptic cleft, reaching maximum levels at about 60 min post-challenge (e.g., de Souza Silva et al., 1997). It is hypothesized that after entrance of the radiolabeled molecules into the synaptic cleft, exgoneous and endogenous ligands compete for D_2_ receptor binding sites. Apparently, this may be visualized by the reduction of [^123^I]IBZM binding from min 75 to min 135 post-challenge in the the present experiment, as well as by the decrease of [^18^F]fallypride and [^11^C]raclopride binding in the studies of Sahin et al. (2014) and Sossi et al. (2009), which were observed from min 30 to min 120 and from min 45 to min 95 post-challenge, respectively.

Since a single application of L-DOPA elicits D_2_ receptor mRNA expression in the neostriatum (Murata and Kanazawa, 1993), it is likely that the later initiation (90 min post-challenge relative to 30–75 min post-challenge) of the imaging study by Opacka-Juffry et al. (1998) provided sufficient time for the development of D_2_ receptor sensitization. Striatal D_2_ receptor supersensitivity also occurs in the unilateral 6-OHDA lesion model (for review see Schwarting and Huston, 1996), and has been demonstrated *in vivo* in both the lesioned and intact contralateral hemisphere (Nikolaus et al., 2003). Thus, the elevation of D_2_ receptor binding observed by Hume et al. (1995) in the ipsilateral as well as contralateral striatum can be accounted for by D_2_ receptor sensitization. In 6-OHDA lesioned rats, D_2_ receptor sensitization may occur as a compensatory response to the decreased availability of DA in the lesioned and—to a lesser extent—in the contralateral striatum. In healthy rats, however, the increased availability of DA induced by L-DOPA may be assumed to elicit a compensatory desensitization of D_2_ receptor binding sites, leading to a lowered inhibitory input to the target regions of DAergic neurons. DA is known to inhibit the release of GABA via D_2_ receptor action (Girault et al., 1986), while GABA, in turn, inhibits the release of DA (Grace and Bunney, 1979). Thus, decreased inhibitory input to GABAergic neurons is likely to decrease striatal DA efflux, leading to a compensatory upregulation of D_2_ receptor binding sites. Apparently, this delayed effect of L-DOPA occurs when D_2_ receptor imaging is initiated as late as 90 min post-challenge, whereas the earlier effect of L-DOPA is uncovered starting from 30 to 75 min post-challenge. It may be that in 6-OHDA-lesioned rats imaged in the latter time window, the earlier L-DOPA effect normally leading to a reduction of radioligand binding becomes partially masked by the lesion-induced D_2_ receptor sensitization. This is evidenced by the fact that the decrease of D_2_ receptor binding after L-DOPA observed by Sahin et al. (2014) and Sossi et al. (2009) is one order of magnitude lower compared to our findings.

### Rat behavior–median differences and *t–b* curves

Inspection of total time median values (Figures 5–9) show that median ambulation frequency was lower and median sitting duration was higher compared to saline after the higher L-DOPA dose, while median duration and frequency of head-shoulder motility were lower compared to saline after both L-DOPA doses. These results are consistent with previous reports of reduced behavioral activity after administration of the DA precursor molecule in moderate doses to mature animals (McDevitt and Setler, 1981; Grigoriadis et al., 1996).

The analysis of *t–b* curves (Figures 5–9) yielded significant differences between (1) ambulation frequency after 10 mg/kg L-DOPA/benserazide and saline, (2) ambulation frequency after 10 and 5 mg/kg L-DOPA/benserazide, (3) rearing frequency after 10 and 5 mg/kg L-DOPA benserazide, (4) duration of head-shoulder motility after 5 mg/kg L-DOPA benserazide and saline, and, finally, (5) frequency of head-shoulder motility after 10 mg/kg L-DOPA benserazide and saline. Thereby, ambulation frequency curves yielded a higher rate constant after 10 mg/kg L-DOPA relative to both saline and the 5 mg/kg dose, and a higher plateau after 10 mg/kg L-DOPA relative to 5 mg/kg L-DOPA, indicating a *faster* rate of decrease relative to saline, and a *faster* rate of decrease to a *higher* final level relative to the lower L-DOPA dose. Rearing frequency curves showed both a higher rate constant and a higher plateau after 5 relative to 10 mg/kg L-DOPA, suggesting a *higher* rate of decrease to a *higher* final level compared to the higher L-DOPA dose. Analysis of head-shoulder motility duration curves yielded lower linear and quadratic terms after 5 mg/kg L-DOPA/benserazide relative to saline, indicating a *slower* rate of decrease from a *lower* maximum level. Analysis of head-shoulder motility frequency curves evidenced a lower slope and a lower y-intercept after 10 mg/kg L-DOPA/benserazide relative to saline, which also suggests a *slower* rate of decrease from a *lower* maximum level.

We use the method of fitting mathematical models to the individual behavioral parameters, employing either linear or non-linear regression analysis. The benefit of this approach is the gaining of information on the temporal dynamics of behavior, which is not achieved by the standard dose-response curves relating changes in behavior merely to dose but not to time. In the present study, the same mathematical models as in our previous investigation (Nikolaus et al., 2014b) could be fit to the individual behavioral parameters, which underlines the general suitability of the *t–b* curve analysis approach.

The present results largely agree with our previous findings on motor/exploratory behaviors after challenge with saline and either 5 or 10 mg/kg L-DOPA/benserazide. The most important consistency is the finding of significantly reduced duration and frequency of head-shoulder motility after both L-DOPA doses. However, in the present study, the *t–b* curves of duration of head-shoulder motility depicted no increase of behavior to the maximum, but merely the subsequent decrease. Consequently, we cannot infer a slower rate of increase in duration of head-shoulder motility as found in the previous study, but, instead, a slower rate of decrease from a lower maximum level. *t–b* Curves of frequency of head-shoulder motility were similar in both studies, showing a slower rate of decrease of frequency of head-shoulder motility from a lower maximum level.

The findings on frequency of ambulation also agree in both studies, showing a significant reduction of ambulation frequency after the higher L-DOPA dose relative to saline. The *t–b* curves of ambulation frequency were also similar in both studies, indicating a faster rate of decrease after 10 mg/kg L-DOPA/benserazide relative to saline, and a faster rate of decrease to a higher final level relative to the lower L-DOPA dose.

On the other hand, in the present study the effects of L-DOPA on duration of sitting were less obvious. While in the previous study a significantly higher sitting duration and faster rate of increase of sitting duration to a higher final level was found after both L-DOPA doses relative to saline, in the present study, a significantly higher median sitting duration was merely observed after the higher L-DOPA dose.

As in the previous study, the reduction of head-shoulder motility after both L-DOPA doses relative to saline became evident in the second time frame (min 6–10). But contrary to the precedent results, significant decreases of ambulation frequency as well as increases of sitting duration relative to saline were detected already in the first time frame (min 1–5). This coincides with the onset of measurable DA release, which in *in vivo* microdialysis studies was observed already at the moment of i.p. injection of L-DOPA/benserazide (de Souza Silva et al., 1997), suggesting an association between the reduction of motor/exploratory activity and the increased availability of endogenous DA.

Taken together, also the present results show a time-dependent decline of motor and exploratory behaviors in the open field. The reduction of motor and exploratory activies such as ambulation and head-shoulder motility over time is generally considered to reflect behavioral habituation (for review see Leussis and Bolivar, 2006). Since these behavioral changes are likely to be related to the L-DOPA-induced increase in endogenous DA, and DA is known to be involved in learning and memory (for review see Myhrer, 2003), the reduction in behavioral markers of activity after L-DOPA, also in the present study might be due to its action on the rate of behavioral habituation to a novel environment.

### Correlation analysis

After 5 mg/kg L-DOPA/benserazide, D_2_ receptor binding correlated negatively with sitting frequency (min 21–25). After 10 mg/kg L-DOPA/benserazide, D_2_ receptor binding correlated positively with the frequency of head-shoulder motility (min 1–30), grooming frequency (min 11–15, min 1–30), and sitting duration (min 25–30).

Thus, lower D_2_ receptor binding was associated with a decrease in exploratory behavior (head-shoulder motility) and a decrease of grooming throughout the whole testing time, but an increase in sitting frequency at the end of the trial. Since the reduction of striatal D_2_ receptor binding after L-DOPA reflects an increased availability of DA, it follows that a higher amount of neostriatal DA decreased exploratory and grooming activity, but facilitated sitting behavior, at least at the end of the trial, when, interestingly, the animals sat down more often but in total for a shorter time.

In our previous study (Nikolaus et al., 2014b), DAT binding correlated negatively with sitting frequency (min 11–15), and positively with both duration and frequency of head-shoulder motility (min 16–20) after 5 mg/kg L-DOPA/benserazide. Morever, in contrast to D_2_ receptor binding, DAT binding correlated positively with duration (min 26–30) and frequency (min 21–25) of rearing as well as with duration of head-shoulder motility (min 21–25) after saline. Since the reduction of striatal DAT binding after L-DOPA can be assumed to reflect increased availability of DA, it may be conjectured—in analogy to the results obtained by D_2_ receptor imaging—that a higher amount of neostriatal DA decreased motor/exploratory behaviors and facilitated sitting. Strikingly, however, DAT imaging in contrast to D_2_ receptor imaging implies that this relation holds for both L-DOPA and saline.

If striatal DA levels rise, more DA molecules may bind to striatal D_2_ receptors (leading to a reduction of [^123^I]IBZM binding). Concomitantly, free DA is transported back into the presynaptic terminal via DAT binding sites (leading to the reduction of [^123^I]FP-CIT binding in the precedent investigation). Apparently, with the [^123^I]FP-CIT-DAT imaging approach, the association between behavior and DA molecules binding to the DAT can be already detected, when DA is not supposed to rise beyond “normal” levels as is the case after pre-treatment with saline. This is not the case with the [^123^I]IBZM-D_2_ receptor imaging approach.

[^123^I]IBZM-SPECT does not allow to differentiate between D_2_ autoreceptor and D_2_ heteroreceptor binding sites. If DA levels rise, DA molecules bind to either site reducing the possibility of both pre- and postsynaptic receptor binding for [^123^I]IBZM. The critical issue, however, appears to be that DA not only triggers feedback inhibition at the presynaptic D_2_ autoreceptor binding sites and desensitizises postsynaptic D_2_ heteroreceptors, but—in response to the D_2_ autoreceptor-induced reduction of DA release—also leads to a renewed sensitization of postsynaptic binding sites. Possibly, the renewed sensitization of postsynaptic D_2_ receptors binding sites hampers the visualization of altered [^123^I]IBZM binding in relation to alterations of motor/exploratory behaviors, if DA levels are within the “normal” range. If this range is exceeded, as may be assumed for L-DOPA challenge, the balance between DA concentration and receptor regulation state is likely to become unhinged, and the increased availability of DA—in the given time window—can no longer be masked by the upregulation of D_2_ receptor binding sites.

Furthermore, it is interesting that motor/exploratoy behaviors are correlated with D_2_ receptor binding after both L-DOPA doses, whereas no correlations were observed between motor/exploratory behaviors and DAT binding after 10 mg/kg. A previous study had indicated a biphasic action of L-DOPA with a higher effect on DAT after the lower dose of L-DOPA (Nikolaus et al., 2013). It may be that DA concentrations after 10 mg/kg L-DOPA at the time between application and equilibration of [^123^I]FP-CIT were lower compared to the 5 mg/kg dose, because the higher dose may have promoted the release of DA in concentrations sufficient to activate feedback inhibition at the presynaptic terminal. The shorter time span between the application of L-DOPA and the equilibration of the D_2_ receptor radioligand may have precluded the occurrence of biphasic actions in the [^123^I]IBZM-D_2_ receptor imaging approach. Further investigations are needed, which explicitly address the issue of suitability of either approach in terms of both L-DOPA dosage and time between pharmacological challenge and initiation of SPECT data acquisition.

## Conclusions

The present complementary investigation of D_2_ receptor binding and behavioral parameters yielded reductions of striatal D_2_ receptor binding after L-DOPA, which reflect increased availability of endogenous DA. As L-DOPA-treated animals displayed less ambulation, less head-shoulder motility and more sitting than saline-treated animals, it may be assumed that the decrease of behavioral activity is associated with the increased availibility of DA.

The analysis of *t–b* curves for ambulation frequency evidenced a *faster* rate of decrease after 10 mg/kg L-DOPA/benserazide relative to saline, and a *faster* rate of decrease to a *higher* final level relative to the lower L-DOPA dose. Rearing frequency curves yielded a *higher* rate of decrease to a *higher* final level after 5 mg/kg L-DOPA/benserazide compared to the higher L-DOPA dose. Analysis of *t–b* curves for both duration and frequency of head-shoulder motility indicated a *slower* rate of decrease of duration and frequency of motility from a *lower* maximum level after 5 mg/kg and 10 mg/kg L-DOPA/benserazide, respectively, relative to saline. Our results largely agree with previous findings (Nikolaus et al., 2014b) and suggest that decline of behavioral markers of activity after L-DOPA may be due to its action on the rate of behavioral habituation to a novel environment. The moderate association between D_2_ receptor binding and parameters of motor and exploratory behavior as evidenced by correlation analysis is in agreement with previous evidence on the relevance of brain regions beyond the neostriatum (such as the hippocampus and the prefrontal cortex) for the unfolding of central L-DOPA action (Navailles et al., 2010). It is also consistent with the role assigned to neurotransmitters beyond DA (such as serotonin, acetylcholine, and glutamate) for motor activity as well as habituation learning (e.g., Dringenberg et al., 1995; Carey et al., 1998; Schildein et al., 2002).

## Author contributions

Experimental design: SN, JH, MASS and HWM. Performance of imaging and behavioral studies: SN. Evaluation and statistical analysis of imaging and behavioral studies: SN and MB. Interpretation of findings: SN, JH, MASS and HWM. Writing and editing of the manuscript: SN, JH, MASS, HWM, HH, CM and CA.

### Conflict of interest statement

The authors declare that the research was conducted in the absence of any commercial or financial relationships that could be construed as a potential conflict of interest.
