# Corrigendum: Relationship Between L-DOPA-Induced Reduction in Motor and Exploratory Activity and Striatal Dopamine D_2_ Receptor Binding in the Rat

**DOI:** 10.3389/fnbeh.2016.00036

**Published:** 2016-03-15

**Authors:** Susanne Nikolaus, Markus Beu, Maria A. de Souza Silva, Joseph P. Huston, Hubertus Hautzel, Claudia Mattern, Christina Antke, Hans-Wilhelm Müller

**Affiliations:** ^1^Clinic of Nuclear Medicine, University Hospital DüsseldorfDüsseldorf, Germany; ^2^Center for Behavioural Neuroscience, Institute of Experimental Psychology, Heinrich-Heine UniversityDüsseldorf, Germany; ^3^M et P Pharma AGEmmetten, Switzerland; ^4^Oceanographic Center, Nova Southeastern UniversityFort Lauderdale, FL, USA

**Keywords:** D_2_ receptor, [^123^I]IBZM, L-DOPA methylester, motor behavior, exploratory behavior, habituation, time-behavior curves, small animal SPECT

Line 13–19 on p. 5, right column, should read as follows:

“D_2_ receptor binding relative to baseline amounted to 21 ± 35% after 5 mg/kg L-DOPA/benserazide and to 19 ± 15% after 10 mg/kg L-DOPA/benserazide. Multiplication of percentual decreases by K_exo_/K_endo_ yielded mean increases of synaptic DA by 213 ± 360% after 5 mg/kg L-DOPA/benserazide and by 199 ± 160% after 10 mg/kg L-DOPA/benserazide.”

Likewise, l. 9–14 on p. 13, right column, should read as follows:

“Changes of synaptic DA concentrations were estimated by multiplying the percentual alteration of exogenous ligand binding with the ratio of affinities (K_exo_/K_endo_), resulting in mean increases of DA by 213 ± 360% after 5 mg/kg and to 199 ± 160% after 10 mg/kg L-DOPA/benserazide.”

This error does not change the scientific conclusions of the article in any way.

## Author contributions

Writing of the manuscript: SN. Editing of the manuscript: MB, MASS, JPH, HH, CM, CA, and HWM.

### Conflict of interest statement

The author declares that the research was conducted in the absence of any commercial or financial relationships that could be construed as a potential conflict of interest.

